# How to Partition a Quantum Observable

**DOI:** 10.3390/e26070611

**Published:** 2024-07-20

**Authors:** Caleb Merrick Webb, Charles Allen Stafford

**Affiliations:** Department of Physics, University of Arizona, Tucson, AZ 85721, USA; staffordphysics92@gmail.com

**Keywords:** open quantum system, entropy, partitioning, thermodynamics

## Abstract

We present a partition of quantum observables in an open quantum system that is inherited from the division of the underlying Hilbert space or configuration space. It is shown that this partition leads to the definition of an inhomogeneous continuity equation for generic, non-local observables. This formalism is employed to describe the local evolution of the von Neumann entropy of a system of independent quantum particles out of equilibrium. Crucially, we find that all local fluctuations in the entropy are governed by an entropy current operator, implying that the production of entanglement entropy is not measured by this partitioned entropy. For systems linearly perturbed from equilibrium, it is shown that this entropy current is equivalent to a heat current, provided that the system-reservoir coupling is partitioned symmetrically. Finally, we show that any other partition of the coupling leads directly to a divergence of the von Neumann entropy. Thus, we conclude that Hilbert-space partitioning is the only partition of the von Neumann entropy that is consistent with the laws of thermodynamics.

## 1. Introduction

Applying the laws of thermodynamics to open quantum systems begs the question of how to partition quantum observables between the system and its environment. In particular, the off-diagonal operators describing the interaction of a system and its environment define an interface, and it is an open question of how to divide that interface between the system and the environment when computing thermodynamic quantities. Various schemes have been proposed in the literature [1,2,3,4,5,6,7,8], many of which involve assigning part of the interfacial energy to the system and the remainder to the environment. We show that entropy is only well-defined under a Hilbert-space partition, which divides the system-environment coupling equally between the system and the environment.

Consider a global Hilbert space ℋ, which contains all single-particle degrees of freedom available to the universe. From this, we construct the Fock space F(ℋ), within which all many-body theories are described. In the study of open quantum systems, one typically divides the universe into complementary sets of orthogonal, single-particle states $ℋ={ℋ}_{S}⊕{ℋ}_{R}$ describing the subsystem of interest ${ℋ}_{S}$ and a thermal reservoir ${ℋ}_{R}$. The Fock space then decomposes as the familiar product $F(ℋ)≅F({ℋ}_{S})⊗F({ℋ}_{R})$.

Denote L(F(ℋ)) by the set of linear operators acting on F(ℋ). Any observable $\hat{𝒪}\in L(F(ℋ))$ may, of course, be written in the form $\hat{𝒪}={\hat{𝒪}}_{S}+{\hat{𝒪}}_{R}+{\hat{𝒪}}_{SR}$, where ${\hat{𝒪}}_{S/R}\in L(F({ℋ}_{S/R}))$ and ${\hat{𝒪}}_{SR}$ describes the coupling between the two subsystems. One may then ask whether the expectation value $\langle \hat{𝒪}\rangle$ can be sensibly divided into system and reservoir contributions. In particular, what fraction of the averaged coupling term $\langle {\hat{𝒪}}_{SR}\rangle$ should be assigned to each subsystem? For a partitioning of ℋ into subsystems of comparable size, it seems intuitive that the coupling should be partitioned symmetrically, assigning $\frac{1}{2}\langle {\hat{𝒪}}_{SR}\rangle$ to each subsystem. However, in the context of the thermodynamics of open quantum systems, an answer to this question has remained elusive due to subtleties in the distinction between system internal energy and heat [1,2,3,4,5,6,7,8]. This thermodynamic bookkeeping typically falls into one of two camps: either half of the coupling Hamiltonian is assigned to the system, as in the symmetric partition, or all of it is. The remaining fraction is then assigned to the reservoir and used to describe heat dissipated.

In this article, we maintain that any partition of the energetics between two subsystems should be inherited from the division of the single-particle states $ℋ\to {ℋ}_{S}⊕{ℋ}_{R}$. Moreover, it will be shown that such a “Hilbert-space partition” of any observable leads directly to the symmetric partition.

Of central interest in the study of open quantum systems is the von Neumann entropy $S=\mathrm{Tr}\{-\hat{\rho }\ln\hat{\rho }\}$, where $\hat{\rho }$ is the density matrix describing the state of the global system in F(ℋ), and throughout this paper $\hbar =k_{B}=1$. A description of the *system entropy* may be constructed by means of the reduced density matrix, defined from $\hat{\rho }$ by tracing out the reservoir degrees of freedom, ${\hat{\rho }}_{S}^{red}={\mathrm{Tr}}_{R}\hat{\rho }$. The entropy of the reduced state of the system is then defined as $S_{S}^{red}=-\mathrm{Tr}\{{\hat{\rho }}_{S}^{red}\ln({\hat{\rho }}_{S}^{red})\}$. Using a quantum master equation to describe the dynamics of the reduced density matrix for the system, it has been shown [9,10] that this entropy obeys

(1) $$\begin{array}{c} \frac{d}{dt}S_{S}^{red}=\frac{J_{Q}}{T}+\frac{d}{dt}\Sigma , \end{array}$$

where $J_{Q}$ is a heat current flowing between the system and reservoir, and the entropy production Σ(t) denotes the mutual information or the information that is lost when neglecting correlations between the subsystems. While interesting from the point of view of quantum information theory, this production of entropy, we argue, is not thermodynamic in character.

As a simple illustration of our argument, consider as a subsystem a single site within an infinite, fermionic, tight-binding chain in thermal equilibrium at temperature *T*,

(2) $$\begin{array}{cc} \hat{H} & =-t\sum_{n}{\hat{c}}_{n}^{†}{\hat{c}}_{n+1}+h.c., \end{array}$$

(3) $$\begin{array}{cc} \hat{\rho } & =\frac{e^{-\beta \hat{H}}}{\mathrm{Tr}\{e^{-\beta \hat{H}}\}}. \end{array}$$

Here, we have taken the chemical potential to lie in the center of the electron band. With the chain in thermal equilibrium, one might expect that all extensive quantities should be uniformly distributed throughout the chain. In particular, for the thermodynamic entropy of a single site, we anticipate that $S=\frac{1}{N}S_{total}$, where *N* is the number of sites in the chain and

(4) $$\begin{array}{cc} S_{total} & =-\sum_{k}[f_{k}\ln(f_{k})+\left(1-f_{k}\right)\ln(1-f_{k})], \end{array}$$

with $f_{k}=\frac{1}{e^{\beta \epsilon_{k}}+1}$ the Fermi–Dirac distribution for orbital |k〉 at temperature $T=\beta^{-1}$. On the other hand, the reduced state for a single site will be of the form ${\hat{\rho }}^{red}=f\hat{n}+\left(1-f\right)\left(1-\hat{n}\right)$, where $\hat{n}$ is the number operator for the site and *f* gives the probability that this site is occupied. Using the results presented in Ref. [11], it can be shown that

(5) $$\begin{array}{c} f=\frac{1}{N}\sum_{k}f_{k}=1/2, \end{array}$$

which is to be expected for the occupancy of a single site given μ in the center of the band, regardless of temperature. The entropy of the reduced state is thus

(6) $$\begin{array}{c} S^{red}=-f\ln(f)-\left(1-f\right)\ln(1-f)=\ln(2), \end{array}$$

independent of temperature. Such entropy is to be expected for the chain’s classical counterpart in a microcanonical ensemble, with *N* sites filled by N/2 particles. The classical picture neglects the entanglement between the fermions occupying the chain; though non-interacting, the antisymmetric nature of the fermionic wavefunctions enforces a maximal entanglement for all states in the ensemble. Indeed, upon comparing Equations (4) and (6), we see that $S-S^{red}=\frac{\Sigma }{N}$, with Σ the total entanglement entropy of the lattice sites.

**Figure 1:**
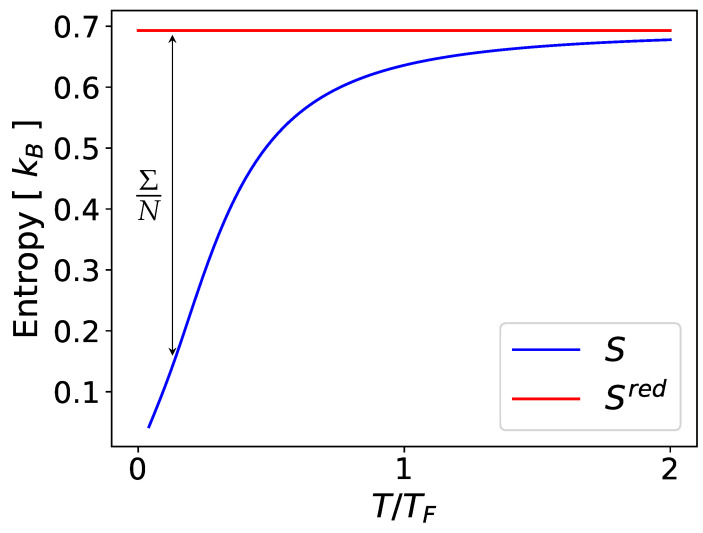
Comparison of entropy density with the entropy of the reduced state of a single site in an infinite tight-binding chain at thermal equilibrium and chemical potential μ=0. The former clearly satisfies the third law of thermodynamics, while the latter is independent of temperature. The difference between the two entropies is given exactly by the entanglement entropy per site of the lattice.

In particular, as T→0, the chain approaches a pure state with all particles maximally entangled. Thus, we should have that $\lim_{T\to 0}S_{total}\left(T\right)=0$, in accordance with the third law of thermodynamics. A comparison between the entropy density $S_{total}/N$ and the entropy of the reduced state is shown in Figure 1.

Clearly, the entropy of the reduced state does not have a meaningful thermodynamic interpretation, whereas the entropy density does, at least for this toy model. In what follows, it will be shown that the Hilbert-space partition can be employed to give a local description of the entropy that *does* have a meaningful thermodynamic interpretation. Importantly, it will be shown that this partitioned entropy does not measure the entanglement between the subsystems.

## 2. Hilbert-Space Partition

In what follows, we will denote by $\hat{𝒪}{|}_{S}$ the Fock-space operator corresponding to the partition of observable $\hat{𝒪}$ over the subspace ${ℋ}_{S}\subset ℋ$ of Hilbert space. We seek a partition that is

1. Inherited from the division of single-particle states on $ℋ={ℋ}_{S}⊕{ℋ}_{R}$, and
2. Additive over subspaces: $\langle \hat{𝒪}\rangle =\langle \hat{𝒪}{|}_{S}\rangle +\langle \hat{𝒪}{|}_{R}\rangle$.

Recall from Section 1 that the structure $F(ℋ)≅F({ℋ}_{S})⊗F({ℋ}_{R})$ of the many-particle Fock space is inherited from that of the underlying single-particle Hilbert space $ℋ≅{ℋ}_{S}⊕{ℋ}_{R}$.

**Figure 2:**
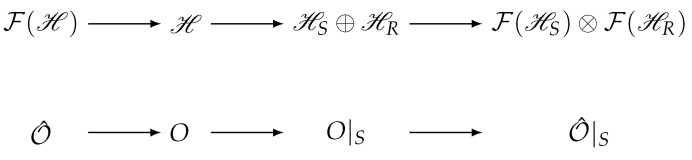
The general scheme for the partitioning of a Fock-space operator $\hat{𝒪}$. First, the matrix *O* in the first quantization is constructed from the matrix elements of $\hat{𝒪}$. A partition is then constructed for ${O\to O|}_{S}$. Finally, this operator is second-quantized to form the partitioned operator, acting on the partitioned Fock Space.

For the sake of clarity, we focus for now on one-body observables $\hat{𝒪}=\sum_{nm}O_{nm}{\hat{c}}_{n}^{†}{\hat{c}}_{m}\in L(F(ℋ))$ and discuss the generalizations to *N*-body observables in Section 6. The matrix elements $O_{nm}$ can be extracted in the form of an observable in first quantization, O∈L(ℋ), which acts on the single-particle Hilbert space. As it is this space that is being divided into system and reservoir states, the partition should be constructed at the level of these matrix elements. By the second quantization of the partitioned operator ${O|}_{S}$, we recover $\hat{𝒪}{|}_{S}\in L(F({ℋ}_{S})⊗F({ℋ}_{R}))$. This construction is outlined in Figure 2.

For the partition of *O*, we define

(7) $$\begin{array}{c} {O|}_{S}\equiv \{\frac{1}{2}O,P_{S}\}, \end{array}$$

where $P_{S}=\sum_{\phi_{j}\in {ℋ}_{S}}|\phi_{j}\rangle \langle \phi_{j}|$ is an orthogonal projector onto ${ℋ}_{S}$, and the anti-commutator {·,·} is included to ensure that ${O|}_{S}$ is Hermitian. The Fock-space operator $\hat{𝒪}{|}_{S}$ is then given by

(8) $$\begin{array}{c} \hat{𝒪}{|}_{S}\equiv \sum_{nm}\langle n|O{|}_{S}|m\rangle {\hat{c}}_{n}^{†}c_{m}. \end{array}$$

That this partition satisfies the additivity condition is a simple consequence of the fact that $P_{S}+P_{R}=𝟙$, so that $\hat{𝒪}{{|}_{S}+\hat{𝒪}|}_{R}=\hat{𝒪}$.

Furthermore, defining

(9) $$\begin{array}{c} \begin{array}{cc} \; & O_{S}=P_{S}OP_{S},\\ \; & O_{SR}=P_{S}OP_{R}+P_{R}OP_{S},\\ \; & O_{R}=P_{R}OP_{R}, \end{array} \end{array}$$

we see that ${O|}_{S}=O_{S}+\frac{1}{2}O_{SR}$. Performing second quantization, one therefore finds that this partition is equivalent to the symmetric partition, wherein the system-reservoir coupling is partitioned equally between subsystems

(10) $$\hat{𝒪}{|}_{S}={\hat{𝒪}}_{S}+\frac{1}{2}{\hat{𝒪}}_{SR}.$$

In the remainder of this paper, we will use $\hat{H}$ to denote the second quantization of an operator H∈L(ℋ).

## 3. Time Dependence

In order to construct the local dynamics of a partitioned expectation value $\langle \hat{𝒪}{|}_{S}\rangle (t)$, it will be useful to consider the Heisenberg evolution of the partitioned operators themselves. In what follows, we consider only non-interacting theories. Even in this simple case, there is, however, some ambiguity in constructing this evolution: should one first evolve the operator $\hat{𝒪}\left(t\right)$ forward in time, and then construct the partition, $\hat{𝒪}{\left(t\right)|}_{S}$? Or partition $\hat{𝒪}\left(0\right)$ and evolve this forward in time, $\hat{𝒪}{|}_{S}\left(t\right)$? Contrary to what one may expect, the two approaches are not generally equivalent.

**Proposition** **1.** *$\hat{𝒪}\left(t\right){{|}_{S}=\hat{𝒪}|}_{S}\left(t\right)$ if and only if $[H,P_{S}]=0$.*

**Proof.**  In the absence of interactions, we have in general that ${\hat{U}}^{†}\hat{𝒪}\hat{U}=\sum_{nm}\langle n|U^{†}OU|m\rangle {\hat{c}}_{n}^{†}{\hat{c}}_{m}$, where $\hat{U}$ is the usual evolution operator generated by Hamiltonian $\hat{H}$. Then, the partition of the time-evolved operator is

(11) $$\begin{array}{c} \hat{𝒪}{\left(t\right)|}_{S}=\frac{1}{2}\sum_{nm}\langle n|\{O_{H}\left(t\right),P_{S}\}|m\rangle {\hat{c}}_{n}^{†}{\hat{c}}_{m}, \end{array}$$

with $O_{H}\left(t\right)=U^{†}OU$ the Heisenberg evolution of *O*. On the other hand, the time evolution of the partitioned operator gives

(12) $$\begin{array}{c} \hat{𝒪}{|}_{S}\left(t\right)=\frac{1}{2}\sum_{nm}\langle n|U^{†}\{O,P_{S}\}U|m\rangle {\hat{c}}_{n}^{†}{\hat{c}}_{m}. \end{array}$$

Clearly, then, these two expressions will only be equivalent if $[H,P_{S}]=0$ so that $U^{†}P_{S}U=P_{S}$. □

In order to preserve the equivalence between the Schrödinger and Heisenberg pictures, one must use Equation (12) to describe the dynamics of the partitioned observable. We emphasize that, in general, $[H,P_{S}]\neq 0$, and so contrary to what one may expect, the evolution of the partition is not given by the partitioning of $\hat{𝒪}\left(t\right)$.

The operator ${𝒪|}_{S}$, therefore, obeys the inhomogeneous continuity equation

(13) $$\begin{array}{c} \frac{d}{dt}{𝒪|}_{S}=J_{𝒪}{{|}_{S}+\Sigma_{𝒪}|}_{S}. \end{array}$$

With

(14) $$\begin{array}{cc} J_{𝒪}{|}_{S} & =\frac{1}{2}\{O_{H},{J|}_{S}\}, \end{array}$$

(15) $$\begin{array}{cc} {J|}_{S} & =i[H,P_{S}],\mathrm{and} \end{array}$$

(16) $$\begin{array}{cc} \Sigma_{𝒪}{|}_{S} & =\frac{1}{2}\{\frac{d}{dt}O_{H},P_{S}\}. \end{array}$$

In the above, the time dependence of the projection operator $P_{S}\left(t\right)=U^{†}P_{S}U$ has been suppressed. Using the fact that the second quantization of a commutator is the commutator of the second quantized operators, it can readily be shown that $\hat{J}{|}_{S}=\frac{d}{dt}{\hat{N}}_{S}$, where ${\hat{N}}_{S}$ is the number operator for subsystem $F({ℋ}_{S})$, implying that ${J|}_{S}$ is a probability current operator. Thus, we interpret $J_{𝒪}{|}_{S}$ as a current operator for the transport of 𝒪 into the subspace ${ℋ}_{S}$. Equation (16) can be written $\Sigma_{𝒪}{{|}_{S}=\left[\frac{d}{dt}𝒪\right]|}_{S}$, and so we interpret this term as the local production of 𝒪 within the subspace ${ℋ}_{S}$. The Fock-space operator $\hat{𝒪}{|}_{S}$, therefore, obeys a similar continuity equation

(17) $$\begin{array}{c} \frac{d}{dt}\hat{𝒪}{|}_{S}={\hat{J}}_{𝒪}{{|}_{S}+{\hat{\Sigma }}_{𝒪}|}_{S}. \end{array}$$

As an illustrative application of this partition, consider the energy current passing through site |i〉 in the tight-binding model

(18) $$\begin{array}{c} \hat{H}=-t\sum_{i,\delta }{\hat{c}}_{i}^{†}{\hat{c}}_{i+\delta }+h.c., \end{array}$$

where the sum on δ denotes a sum over nearest neighbors. Using Equation (14), we find for the energy current flux

(19) $$\begin{array}{c} {\hat{J}}_{H}{|}_{|i\rangle }=\frac{it^{2}}{2}\sum_{\delta ,\delta^{\prime }}{\hat{c}}_{i+\delta +\delta^{\prime }}^{†}{\hat{c}}_{i}-{\hat{c}}_{i}^{†}{\hat{c}}_{i-\delta -\delta^{\prime }}, \end{array}$$

which implies the textbook definition [12] for the energy current operator:

(20) $$\begin{array}{c} {\hat{\vec{J}}}_{H}{|}_{|i\rangle }=-\frac{it^{2}}{2}\sum_{\delta ,\delta^{\prime }}(\vec{\delta }+{\vec{\delta }}^{\prime })c_{i+\delta +\delta^{\prime }}^{†}c_{i}. \end{array}$$

## 4. Density of One-Body Observables

Up until now, we have only discussed partitions over a discrete subspace of ℋ. The same construction can be applied also to subsets $S\subset R^{n}$ by replacing the projector $P_{S}$ with the operator |x〉〈x| for $x\in R^{n}$. We thus define the density of a one-body observable $\hat{𝒪}$ in much the same way as before:

(21) $$\begin{array}{c} \rho_{𝒪}\left(x\right)=\frac{1}{2}\{O,|x\rangle \langle x|\} \end{array}$$

and

(22) $$\begin{array}{c} {\hat{\rho }}_{𝒪}\left(x\right)=\sum_{nm}\langle n|\rho_{𝒪}\left(x\right)|m\rangle {\hat{c}}_{n}^{†}{\hat{c}}_{m}. \end{array}$$

Note that, as with the discrete partition, $\hat{𝒪}$ need not be diagonal in position representation to define ${\hat{\rho }}_{𝒪}\left(x\right)$. Rather, we argue that ${\hat{\rho }}_{𝒪}\left(x\right)$ describes the influence of the non-local observable $\hat{𝒪}$ at the location $x\in R^{n}$.

This interpretation may be clarified by considering the operator $\hat{𝒪}$ in position representation

(23) $$\begin{array}{cc} \hat{𝒪} & =\int dx\;dy\;\hat{𝒪}\left(x,y\right),\mathrm{with} \end{array}$$

(24) $$\begin{array}{cc} \hat{𝒪}\left(x,y\right) & =\sum_{nm}\left[O_{nm}\psi_{n}\left(x\right)\psi_{m}^{*}\left(y\right)\right]{\hat{\psi }}^{†}\left(x\right)\hat{\psi }\left(y\right), \end{array}$$

where ${\hat{\psi }}^{†}\left(x\right),\hat{\psi }\left(x\right)$ are the usual fermionic field creation and annihilation operators, and $\psi_{n}\left(x\right)=\langle x|n\rangle$. The density in Equation (22) can be rewritten in terms of $\hat{𝒪}\left(x,y\right)$ as

(25) $$\begin{array}{c} {\hat{\rho }}_{𝒪}\left(x\right)=\frac{1}{2}\int dy(\hat{𝒪}\left(x,y\right)+\hat{𝒪}\left(y,x)\right). \end{array}$$

Following the same arguments as in the previous section, we find that the density operator obeys a continuity equation

(26) $$\begin{array}{c} \frac{d}{dt}{\hat{\rho }}_{𝒪}\left(x,t\right)=-\nabla \cdot {\hat{\vec{j}}}_{𝒪}\left(x,t\right)+{\hat{\sigma }}_{𝒪}\left(x,t\right), \end{array}$$

where

(27) $$\begin{array}{cc} {\vec{j}}_{𝒪}\left(x,t\right) & =\frac{1}{2}\{O_{H},\vec{j}\left(x,t\right)\}, \end{array}$$

(28) $$\begin{array}{cc} \vec{j}\left(x,t\right) & =\frac{1}{2m}U^{†}\{\vec{p},|x\rangle \langle x|\}U, \end{array}$$

(29) $$\begin{array}{cc} \sigma_{𝒪}\left(x,t\right) & =\frac{1}{2}\{\frac{d}{dt}O_{H},U^{†}|x\rangle \langle x|U\}. \end{array}$$

In the above, $\vec{p}=-i\nabla$ is the usual momentum operator. Moreover, in slight contrast to Equations (15) and (16), we have made explicit the time evolution of the projection operator $U^{†}|x\rangle \langle x|U$. The definitions of current density in Equations (27) and (28) are consequences of the following claim:

**Proposition** **2.** *$i[H,|x\rangle \langle x|]=-\nabla \cdot \vec{j}\left(x\right)$, where $\vec{j}\left(x\right)=\frac{1}{2m}\{\vec{p},|x\rangle \langle x|\}$.*

**Proof.**  Only the kinetic part of the Hamiltonian is non-vanishing in the commutator, since for any two states |n〉 and |m〉

$$\langle n|[V,|x\rangle \langle x|]|m\rangle =\psi_{m}\left(x\right)V\psi_{n}^{*}\left(x\right)-\psi_{n}^{*}\left(x\right)V\psi_{m}\left(x\right)=0,$$

assuming that *V* acts as a simple multiplication operator in position representation. Then

$$\begin{array}{c} i[H,|x\rangle \langle x|]=\frac{i}{2m}[{\vec{p}}^{\;2},|x\rangle \langle x|]. \end{array}$$

To proceed, we consider the matrix elements

$$\begin{array}{cc} i\langle n|[{\vec{p}}^{\;2},|x\rangle \langle x|]|m\rangle & =\nabla \cdot \psi_{m}{\left(i\nabla \psi_{n}\right)}^{*}+\psi_{n}^{*}\left(i\nabla \psi_{m}\right))\\ \; & =-\nabla \cdot \langle n|\{\vec{p},|x\rangle \langle x|\}|m\rangle . \end{array}$$

Therefore, for any two states |n〉 and |m〉, $\langle n|i[H,|x\rangle \langle x|]|m\rangle =-\frac{1}{2m}\nabla \cdot \langle n|\{\vec{p},|x\rangle \langle x|\}|m\rangle$, which proves the claim. □

Note that $\langle \psi |\vec{j}\left(x,t\right)|\psi \rangle$ gives the usual probability current density of the state |ψ〉.

## 5. Entropy Partitions

Of particular interest is the partition of the von Neumann entropy. Define in the Schrödinger picture the entropy operator

(30) $$\begin{array}{c} \hat{S}\left(t\right)=-\ln\hat{\rho }\left(t\right), \end{array}$$

where $\hat{\rho }\left(t\right)$ is the density matrix for the global ensemble. Although not a quantum observable in the usual sense, it is nonetheless a statistical operator on Fock space whose quantum statistical average gives the von Neumann entropy and which provides a platform to partition entropy in the same way dynamical observables were partitioned in Section 2.

It follows that $S(t)=\langle \hat{S}\left(t\right)\rangle =S(0)$ due to the unitary evolution of $\hat{\rho }$. The Heisenberg picture entropy operator is then ${\hat{S}}_{H}\left(t\right)=-{\hat{U}}^{†}\ln\hat{\rho }\left(t\right)\hat{U}=\hat{S}\left(0\right)$. Therefore, the entropy operator is constant, which is in agreement with our expectation that the global entropy should be constant under unitary evolution.

In the absence of inter-particle interactions, the state of the system may be taken to be a product of the form

(31) $$\begin{array}{c} \hat{\rho }=\prod_{|k\rangle }\left[f_{k}{\hat{c}}_{k}^{†}{\hat{c}}_{k}+\left(1-f_{k}\right){\hat{c}}_{k}{\hat{c}}_{k}^{†}\right], \end{array}$$

where $\left\{|k\rangle \right\}$ is any set of single-particle orbitals that span ℋ, and $f_{k}$ describes the probability that the state |k〉 is occupied. We define the *statistical basis* to be the set of single-particle orbitals over which the density matrix factorizes. Such a state may describe, for example, a quantum system initially in equilibrium that is subsequently acted upon by a time-dependent external force.

For such a product state, the entropy operator becomes a sum of particle- and hole-ordered single-particle operators

(32) $$\begin{array}{c} \hat{S}=-\sum_{k}\ln(f_{k}){\hat{c}}_{k}^{†}{\hat{c}}_{k}-\sum_{k}\ln(1-f_{k}){\hat{c}}_{k}{\hat{c}}_{k}^{†}. \end{array}$$

The arguments of the previous sections hold just as well for a hole-ordered operator, and so the entropy may be partitioned as in Equations (7) and (8). In particular, its partition and density obey the continuity equations

(33) $$\begin{array}{cc} \; & \frac{d}{dt}\hat{S}{{|}_{S}={\hat{J}}_{S}|}_{S}, \end{array}$$

(34) $$\begin{array}{cc} \; & \frac{d}{dt}{\hat{\rho }}_{S}\left(x\right)=-\nabla \cdot {\hat{\vec{j}}}_{S}\left(x\right), \end{array}$$

where

(35) $$\begin{array}{c} {\hat{\rho }}_{S}\left(x\right)=-\frac{1}{2}\sum_{k,k^{\prime }}\psi_{k^{\prime }}\left(x\right)\psi_{k}^{*}\left(x\right)\left[\ln(f_{k}f_{k^{\prime }}){\hat{c}}_{k}^{†}{\hat{c}}_{k^{\prime }}+\ln(\left(1-f_{k}\right)\left(1-f_{k^{\prime }}\right)){\hat{c}}_{k^{\prime }}{\hat{c}}_{k}^{†}\right], \end{array}$$

(36) $$\begin{array}{c} {\hat{\vec{j}}}_{S}\left(x\right)=-\frac{1}{2}\sum_{k,k^{\prime }}\langle k|\;\vec{j}\left(x\right)|k^{\prime }\rangle \left[\ln(f_{k}f_{k^{\prime }}){\hat{c}}_{k}^{†}{\hat{c}}_{k^{\prime }}+\ln(\left(1-f_{k}\right)\left(1-f_{k^{\prime }}\right)){\hat{c}}_{k^{\prime }}{\hat{c}}_{k}^{†}\right] \end{array}$$

are the entropy density operator and the entropy current density operator, respectively. The net entropy current operator ${\hat{J}}_{S}{|}_{S}$ can be obtained as minus the surface integral of Equation (36).

Crucially, as a consequence of global entropy conservation under unitary evolution, we find that there is no local entropy production in Equations (33) and (34). Compared to Equation (1), we interpret this to mean that the entropy partition proposed in this article does not measure entanglement entropy between subsystems. This entropy partition may therefore provide a more faithful description of the local thermodynamic entropy of the system [13].

### 5.1. Evolution of Entropy Density

In this section, we illustrate the continuity equation [Equation (33)] by modeling the evolution of the entropy density in a one-dimensional chain of fermions in the tight-binding model. We consider a finite chain of N=100 sites with zero occupancy and a single, isolated site with a probability of occupancy of f=1/2, which are coupled on the left end of the chain at time t=0. The initial density matrix for this configuration is

(37) $$\begin{array}{c} \hat{\rho }\left(t=0\right)=1/2({\hat{c}}_{k_{0}}^{†}{\hat{c}}_{k_{0}}+{\hat{c}}_{k_{0}}{\hat{c}}_{k_{0}}^{†})\prod_{i=1}^{N}{\hat{c}}_{k_{i}}{\hat{c}}_{k_{i}}^{†} \end{array}$$

where ${\hat{c}}_{k_{0}}^{†}$ (${\hat{c}}_{k_{0}}$) creates (annihilates) a fermion on the initially isolated site, and $\left\{|k_{i}\rangle \right\}$ are eigenstates of the uncoupled chain. The unoccupied sites contribute nothing to the total entropy, and so the entropy operator is

(38) $$\begin{array}{c} \hat{S}=\ln(2)({\hat{c}}_{k_{0}}^{†}{\hat{c}}_{k_{0}}+{\hat{c}}_{k_{0}}{\hat{c}}_{k_{0}}^{†}). \end{array}$$

Using Equation (12) with projection operator $P_{S}=|m\rangle \langle m|$, the entropy density operator for lattice site *m* may be expressed on a statistical basis (see discussion following Equation (31)) as

(39) S^|m=ln(2)∑i=0NRe{〈k0(t)|m〉〈m|ki(t)〉c^k0†c^ki+c^k0c^ki†}.

The entropy density on site *m* is therefore given as

(40) $$\begin{array}{c} \langle \hat{S}{|}_{m}\rangle \left(t\right)={\ln(2)|\langle k_{0}\left(t\right)|m\rangle |}^{2}, \end{array}$$

with evolution governed by the tight-binding Hamiltonian on the coupled lattice

(41) $$\begin{array}{c} \hat{H}=-t_{0}\sum_{n=0}^{N-1}\left[{\hat{c}}_{n}^{†}{\hat{c}}_{n+1}+{\hat{c}}_{n+1}^{†}{\hat{c}}_{n}\right]. \end{array}$$

Here $t_{0}$ is the nearest-neighbor hopping integral.

From Equations (14) and (15) we obtain the following expression for the entropy current density for site *m*

(42) 〈J^S|m〉(t)=2t0ln(2)Im{〈k0(t)|m+1〉〈m|k0(t)〉+〈k0(t)|m−1〉〈m|k0(t)〉}.

**Figure 3:**
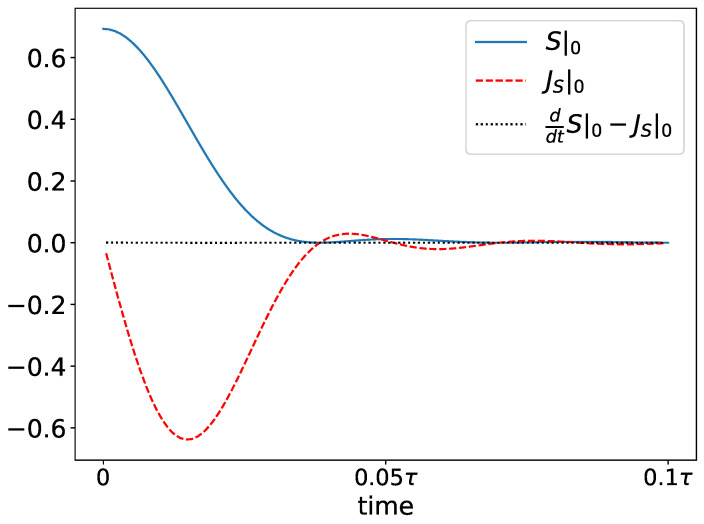
Entropy density and current density on the initially isolated site as a function of time. The entropy density is calculated from Equation (40) and the current from Equation (42).

In Figure 3, we plot the entropy density and entropy current density on the initially uncoupled site (the 0*^th^* site) as a function of time. The entropy density, ${S|}_{0}$, is calculated using Equation (40), and the entropy current density using Equation (42). In the same figure, we also plot the difference $\frac{d}{dt}S{{|}_{0}-J_{S}|}_{0}$ in order to emphasize the local conservation of entropy. The timescale τ is defined as the time required for a wavepacket traveling at the maximum group velocity to traverse the full lattice.

**Figure 4:**
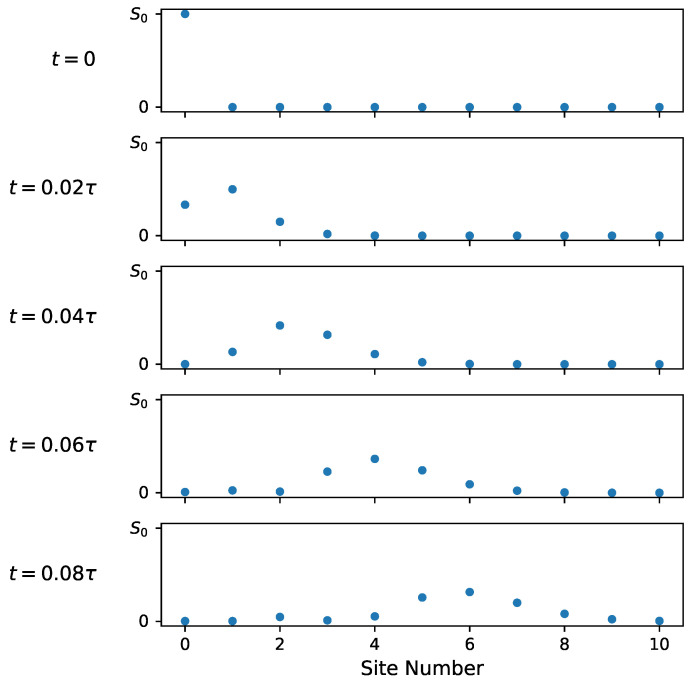
Entropy density on 10 leftmost lattice sites for several moments in time. $S_{0}$ is the initial total entropy of the system (isolated site).

**Figure 5:**
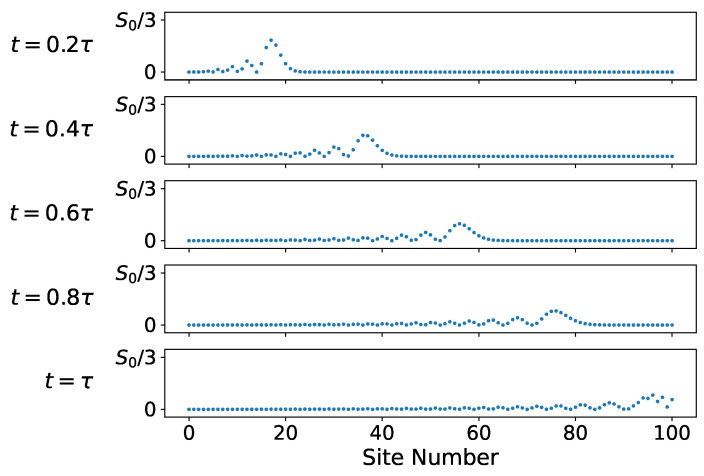
Entropy density on every lattice site for several moments in time. Here the maximum entropy/site displayed is one-third of the initial entropy $S_{0}$.

The evolution of the entropy density on the lattice is explored further in Figure 4 and Figure 5. Figure 4 shows the entropy density evolution on the first ten lattice sites shortly after the 0*^th^* site is coupled to the chain. Figure 5 shows the entropy density on the full lattice over the full timescale τ. Note the change in vertical scale in Figure 5 from $S_{0}$ to $S_{0}/3$. Initially, only the uncoupled site has entropy $S_{0}=\ln(2)$. As the system evolves, this entropy is spread out over the rest of the lattice while maintaining a constant total entropy of $S_{0}$.

### 5.2. Consistency with the Third Law

Applying the Hilbert space partition to the entropy operator leads to a system entropy operator

(43) $$\begin{array}{c} \hat{S}{|}_{S}={\hat{S}}_{S}+\frac{1}{2}{\hat{S}}_{SR}. \end{array}$$

In light of the above discussion, this would imply that the heat added to an open quantum system should be defined as [13]

(44) $$\begin{array}{c} Q_{S}=d\;\langle {\hat{H}}_{S}+\frac{1}{2}{\hat{H}}_{SR}\rangle -\mu d\;\langle {\hat{N}}_{S}\rangle -đW_{S} \end{array}$$

for linear deviations from equilibrium, where ${\hat{N}}_{S}$ is the system number operator and $W_{S}$ is the external work performed on the system.

Various authors [1,2,3,4,6,7,8] have suggested including different fractions of the coupling energy $\langle {\hat{H}}_{SR}\rangle$ in the heat partition. However, it can be shown that any partition of the entropy apart from Equation (43), corresponding to the symmetric partition advocated in Refs. [1,3,4,13], leads to a violation of the third law of thermodynamics. Define the α-partition of $\hat{S}$ as

(45) $$\begin{array}{c} {\hat{S}}^{\alpha }{|}_{S}={\hat{S}}_{S}+\alpha {\hat{S}}_{SR}. \end{array}$$

**Proposition** **3.** *Let $\hat{\rho }$ be a product state of the form Equation (31). Then for any α≠1/2, $\langle {\hat{S}}^{\alpha }{|}_{S}\rangle$ diverges in the limit $f_{k}\to 0,1$ for any k.*

**Proof.**  Define the operators in first quantization $S^{n}=-\sum_{k}\ln(f_{k})|k\rangle \langle k|$ and $S^{p}=-\sum_{k}\ln(1-f_{k})|k\rangle \langle k|$ so that $\hat{S}={\hat{S}}^{n}+{\hat{S}}^{p}$ with ${\hat{S}}^{n}$ and ${\hat{S}}^{p}$ being their corresponding particle- and hole-like quantizations. We will focus our attention on $S^{n}$, anticipating that the treatment for $S^{p}$ will be identical, and drop the superscripts.

The coupling term can therefore be written as the second quantization of the operator

(46) $$\begin{array}{cc} S_{SR} & =P_{S}SP_{R}+P_{R}SP_{S} \end{array}$$

(47) $$\begin{array}{cc} \Rightarrow S^{\alpha }{|}_{S} & =P_{S}SP_{S}+\alpha (P_{S}SP_{R}+P_{R}SP_{S}) \end{array}$$

If we write 1=2α+(1−2α) and make use of $P_{S}+P_{R}=𝟙$, then the last line becomes

(48) $$\begin{array}{c} S^{\alpha }{|}_{S}=\left(1-2\alpha \right)P_{S}SP_{S}+\alpha P_{S}S \end{array}$$

Consider now the matrix elements in the statistical basis, $\left\{|k\rangle \right\}$ (*S* is diagonal in this basis).

(49) $$\begin{array}{c} \langle k|S^{\alpha }{|}_{S}|k\rangle =\left(1-2\alpha \right)\sum_{k^{\prime }}s_{k^{\prime }}{\left|\langle k|P_{S}|k^{\prime }\rangle \right|}^{2}+2\alpha s_{k}\langle k|P_{S}|k\rangle , \end{array}$$

where $s_{k}=-f_{k}\ln(f_{k})-\left(1-f_{k}\right)\ln(1-f_{k})$. We find, then, for the partitioned entropy

(50) $$\begin{array}{cc} \langle {\hat{S}}^{\alpha }{|}_{S}\rangle = & \left(1-2\alpha \right)\sum_{kk^{\prime }}(-f_{k}\ln(f_{k^{\prime }})-\left(1-f_{k}\right)\ln(1-f_{k^{\prime }})){\left|\langle k|P_{S}|k^{\prime }\rangle \right|}^{2}+2\alpha \sum_{k}s_{k}\langle k|P_{S}|k\rangle . \end{array}$$

The first term in Equation (50) diverges if any $f_{k}\in \left\{0,1\right\}$, unless α=1/2 or $\langle k|P_{S}|k^{\prime }\rangle =\delta_{kk^{\prime }}$. The latter condition applies only to a partition of the statistical basis and is certainly not true in general. Thus, we conclude that for a general partition of the entropy of a partially pure state (we define a partially pure state to be any state of the system containing definite occupancies, $f_{k}=0,1$), $\langle {\hat{S}}^{\alpha }{|}_{S}\rangle$ diverges if α≠1/2. □

From Equation (48), we conclude that the only well-defined partition $S^{\alpha }{|}_{S}$ is $S^{1/2}{|}_{S}=\frac{1}{2}\{P_{S},S\}$, the Hilbert-space partition. In particular, this implies that the partitioned entropy of the Gibbs state diverges in the limit T→0 if α≠1/2, since all states above the Fermi level will be unoccupied with definite probability. We conclude that the Hilbert-space partition is the only thermodynamically consistent partition of the entropy and heat for an open quantum system in equilibrium with its surroundings.

Note, though, that our conclusion is even stronger than this. From Equation (50), it is clear that the partitioned entropy will diverge for α≠1/2 if there are *any* localized pure states in the product ensemble Equation (31).

## 6. Many-Body Operators

Until now, we have only considered partitions for one-body observables. It is, however, also possible to construct a partition for *N*-body observables based on the partition of the single-particle Hilbert space. In the remainder of this section, we generalize the above partitions first to 2-body, and then *N*-body observables.

### 6.1. Two-Body Operators

Consider first a two-body operator $O=\sum_{ijkl}A_{ijkl}|ij\rangle \langle kl|$ that acts on the Hilbert space ℋ⊗ℋ. We wish to generalize the partition of *O* onto a subspace ${ℋ}_{S}\subset ℋ$ in such a way that ${O|}_{S}{+O|}_{S^{C}}=O$, where $S^{C}=\{|j\rangle \in ℋ||j\rangle \notin {ℋ}_{S}\}.$ This can be accomplished by adjusting the projection operator as follows $P_{S}\to \frac{1}{2}P_{S}\hat{⊕}P_{S}\equiv \frac{1}{2}(P_{S}⊗𝟙+𝟙⊗P_{S})$:

(51) $$\begin{array}{c} {O|}_{S}\equiv \frac{1}{2}\{O,\frac{P_{S}\hat{⊕}P_{S}}{2}\}. \end{array}$$

Then, because $\left(A\hat{⊕}B)+(C\hat{⊕}D)=(A+C)\hat{⊕}(B+D\right)$ and $P_{S}+P_{S^{C}}=𝟙$, ${O|}_{S}{+O|}_{S^{C}}=\frac{1}{2}\{O,\frac{2𝟙}{2}\}=O$ as needed. It then follows that the partition of the second quantized operator $\hat{𝒪}$ is

(52) $$\begin{array}{c} \hat{𝒪}{|}_{S}=\sum_{ijkl}\langle ij|O{|}_{S}|kl\rangle {\hat{c}}_{i}^{†}{\hat{c}}_{j}^{†}{\hat{c}}_{k}{\hat{c}}_{l}. \end{array}$$

One may generalize the density of observables in the same manner, from which we obtain, in position representation,

(53) $$\begin{array}{cc} {\hat{\rho }}_{𝒪}\left(x\right)=\frac{1}{4}\int & dwdydz[\hat{𝒪}\left(w,x,y,z\right)+\hat{𝒪}\left(x,w,y,z\right)\\ & +\hat{𝒪}\left(w,y,x,z\right)+\hat{𝒪}\left(w,y,z,x\right)]. \end{array}$$

As motivation for this construction, consider the Coulomb interaction

(54) $$\begin{array}{c} \hat{V}=\frac{1}{2}\int dxdy\frac{:\hat{\rho }\left(x\right)\hat{\rho }\left(y\right):}{\left|x-y\right|}, \end{array}$$

where $\hat{\rho }\left(x\right)={\hat{\psi }}^{†}\left(x\right)\hat{\psi }\left(x\right)$, and $:\hat{A}:$ denotes the normal ordering of $\hat{A}$. Defining

$$\hat{V}\left(w,x,y,z\right)=\frac{\delta (w-x)\delta (z-y)}{2|x-y|}({\hat{\psi }}^{†}\left(x\right){\hat{\psi }}^{†}\left(y\right)\hat{\psi }\left(w\right)\hat{\psi }\left(z\right))$$

we see that $\hat{V}=\int dwdxdydz\hat{V}\left(w,x,y,z\right)$. Equation (53) then implies that the energy density is

(55) $$\begin{array}{cc} {\hat{\rho }}_{V}\left(x\right) & =:\hat{V}\left(x\right)\hat{\rho }\left(x\right):,\mathrm{with} \end{array}$$

(56) $$\begin{array}{cc} \hat{V}\left(x\right) & =\frac{1}{2}\int dy\frac{\hat{\rho }\left(y\right)}{\left|x-y\right|}, \end{array}$$

in analogy with the classical description.

### 6.2. N-Body Observables

For the matrix elements of an *N*-body operator $O\in L({ℋ}^{N})$, we define the partition

(57) $$\begin{array}{c} {O|}_{S}=\frac{1}{2}\{O,\frac{1}{N}{\hat{⨁}}_{i=1}^{N}P_{S}\}. \end{array}$$

**Proposition** **4.** *For any N-body observable $\hat{𝒪}$, its partition over ${ℋ}_{S}\subset ℋ$ is given by*

(58) $$\begin{array}{cc} \hat{𝒪}{|}_{S} & =\frac{1}{2N}\{\hat{𝒪},{\hat{N}}_{S}\}-\frac{1}{N}:\hat{𝒪}{\hat{N}}_{S}:,\\ {\hat{N}}_{S} & =\sum_{|n\rangle \in {ℋ}_{S}}{\hat{c}}_{n}^{†}{\hat{c}}_{n}. \end{array}$$

The proof follows straightforwardly from an application of Wick’s theorem to the anticommutator $\frac{1}{2N}\{\hat{𝒪},{\hat{N}}_{S}\}$. Upon comparing the normal ordering to the second quantization of Equation (57), one can see that they differ by exactly $\frac{1}{N}:\hat{𝒪}{\hat{N}}_{S}:$. A detailed proof is given in Appendix A.

**Corollary** **1.** *The expectation value of a partitioned many-body observable $\langle \hat{𝒪}{|}_{S}\rangle$ obeys the continuity equation in Equation (17).*

A detailed proof is given in Appendix B.

Following the same arguments as in Appendix A, one can show that the density of many-body observables is similarly defined.

**Proposition** **5.** *The density of any N-body observable $\hat{𝒪}$ may be defined as*

(59) $$\begin{array}{c} {\hat{\rho }}_{𝒪}\left(x\right)=\frac{1}{2N}\{\hat{𝒪},\hat{\rho }\left(x\right)\}-\frac{1}{N}:\hat{𝒪}\hat{\rho }\left(x\right):. \end{array}$$

## 7. Conclusions

In this article, we have developed a framework to partition quantum observables based on a partition of the underlying single-particle Hilbert space. We have provided explicit expressions for the partition of Fock-space operators corresponding to generic *N*-body observables. For the case of an open quantum system, a bipartite partition between system (S) and reservoir (R) was applied to both the Hamiltonian and the entropy of the system. The Hilbert-space partition was shown to correspond to a symmetric (α=1/2) partition [1,3,4,13] of the off-diagonal 1-body observables, such as the coupling Hamiltonian $H_{SR}$ between system and reservoir. Any partition with α≠1/2 was shown to lead to a singular entropy and hence does not provide a basis to construct consistent thermodynamics from the statistical mechanics of the problem.

## Data Availability

The original contributions presented in the study are included in the article, further inquiries can be directed to the corresponding author.

## Appendix A. Proof of Equation (58)

The proof will require two lemmas. Define by

(A1) $$\begin{array}{c} \begin{array}{cc} Q:L(ℋ) & \to L(F(ℋ))\\ 𝒪 & \mapsto \hat{𝒪} \end{array} \end{array}$$

the map which second quantizes an operator 𝒪.

**Lemma** **A1.**
*Let A and B be two one-body operators. Then*

(A2)
$$\begin{array}{c} Q(\frac{1}{2}\{A,B\})=\frac{1}{2}\{\hat{A},\hat{B}\}-:\hat{A}\hat{B}:. \end{array}$$

**Proof.** 

(A3) $$\begin{array}{c} \begin{array}{cc} \hat{A}\hat{B} & =\sum_{kk^{\prime }}\sum_{qq^{\prime }}A_{kk^{\prime }}B_{qq^{\prime }}{\hat{c}}_{k}^{†}{\hat{c}}_{k^{\prime }}{\hat{c}}_{q}^{†}{\hat{c}}_{q^{\prime }}\\ \; & =\sum_{kq^{\prime }}\left[\sum_{q}A_{kq}B_{qq^{\prime }}\right]{\hat{c}}_{k}^{†}{\hat{c}}_{q^{\prime }}+:\hat{A}\hat{B}:\\ \; & =Q(AB)+:\hat{A}\hat{B}: \end{array} \end{array}$$

Similarly, $\hat{B}\hat{A}=Q(BA)+:\hat{A}\hat{B}:$. The statement of the proof follows immediately from here. □

Using the fact that $Q(P_{S})={\hat{N}}_{S}$ it is readily apparent from this first lemma that the partition of any one-body observable can be constructed as in Equation (58). To show that this holds for any *N*-body observable we will need the following.

**Lemma** **A2.**
*Let*

$$\hat{A}=\sum_{\begin{array}{c} k_{1}\cdots k_{N}\\ k_{N}^{\prime }\cdots k_{1}^{\prime } \end{array}}A_{k_{1}\cdots k_{N}k_{N}^{\prime }\cdots k_{1}^{\prime }}{\hat{c}}_{k_{1}}^{†}\cdots {\hat{c}}_{k_{N}}^{†}{\hat{c}}_{k_{N}^{\prime }}\cdots {\hat{c}}_{k_{1}^{\prime }}\;$$

*be a normal ordered N-body operator and $\hat{B}$ be a one-body operator. Then*

(A4)
$$\begin{array}{c} \left\{ \hat{A},\hat{B}\}=\sum_{\begin{array}{c} k_{1},\cdots ,k_{N}\\ k_{1}^{\prime },\cdots ,k_{N}^{\prime } \end{array}}\;\sum_{j=1}^{N}\begin{array}{cc} & \left[\sum_{q^{\prime }}A_{k_{1}\cdots k_{N}k_{N}^{\prime }\cdots k_{j+1}^{\prime }q^{\prime }k_{j-1}^{\prime }\cdots k_{1}^{\prime }}B_{q^{\prime }k_{j}^{\prime }}+\sum_{q}B_{k_{j}q}A_{k_{1}\cdots k_{j-1}qk_{j+1}\cdots k_{N}k_{N}^{\prime }\cdots k_{1}^{\prime }}\right]{\hat{c}}_{k_{1}}^{†}\cdots {\hat{c}}_{k_{N}}^{†}{\hat{c}}_{k_{N}^{\prime }}\cdots {\hat{c}}_{k_{1}^{\prime }}+2:\hat{A}\hat{B}: \end{array}\right. \end{array}$$

**Proof.** From Wick’s Theorem

(A5) $$\begin{array}{c} \begin{array}{cc} :{\hat{c}}_{k_{N}^{\prime }}\cdots {\hat{c}}_{k_{1}^{\prime }}{\hat{c}}_{q}^{†}{\hat{c}}_{q^{\prime }}: & =\sum_{\begin{array}{c} pairwise\\ contractions \end{array}}:{\hat{c}}_{k_{N}^{\prime }}\cdots \overset{⎴}{{\hat{c}}_{k_{1}^{\prime }}{\hat{c}}_{q}^{†}}{\hat{c}}_{q^{\prime }}:\\ \; & =\sum_{j=1}^{N}\delta_{k_{j}^{\prime }q}{\hat{c}}_{k_{N}^{\prime }}\cdots {\hat{c}}_{k_{j+1}^{\prime }}{\hat{c}}_{q^{\prime }}{\hat{c}}_{k_{j-1}^{\prime }}\cdots {\hat{c}}_{k_{1}^{\prime }}, \end{array} \end{array}$$

and similarly

(A6) $$\begin{array}{c} :{\hat{c}}_{q}^{†}{\hat{c}}_{q^{\prime }}{\hat{c}}_{k_{1}}^{†}\cdots {\hat{c}}_{k_{N}}^{†}:=\sum_{j=1}^{N}\delta_{q^{\prime }k_{j}}{\hat{c}}_{k_{1}}^{†}\cdots {\hat{c}}_{k_{j-1}}^{†}{\hat{c}}_{q}^{†}{\hat{c}}_{k_{j+1}}^{†}\cdots {\hat{c}}_{k_{N}}^{†}. \end{array}$$

The rest follows straightforwardly from here. □

While it seems reasonable that Equation (A2) may be extended to *N*-body observables, one needs to be careful with the normalization. From Lemma A2 with $\hat{B}=\hat{N}$, $B_{ij}=\delta_{ij}$ and so

(A7) $$\begin{array}{cc} \left\{\hat{A},\hat{N}\right\} & =2\sum_{i}^{N}\hat{A}+2:\hat{A}\hat{N}:. \end{array}$$

Therefore

$$\frac{1}{2}\{\hat{A},\hat{N}/N\}-:\hat{A}\hat{N}/N:=\hat{A},$$

confirming that the additional factor of 1/N in Equation (58) correctly normalizes the partition.

Finally, it remains to be shown that Equation (58) follows from the partition defined in Equation (57).

**Proof.** Using Lemma A2 with $\hat{B}={\hat{N}}_{S}$ we recognize the first term in brackets in Equation (A4) as the matrix element

(A8) $$\begin{array}{c} \langle k_{1}\cdots k_{N}|A\left\{|k_{1}^{\prime }\cdots k_{j-1}^{\prime }\rangle ⊗(P_{S}|k_{j}^{\prime }\rangle )⊗|k_{j+1}^{\prime }\cdots k_{N}^{\prime }\rangle \right\}=\langle k_{1}\cdots k_{N}\left|A({𝟙}_{j-1}⊗P_{S}⊗{𝟙}_{N-j})\right|k_{1}^{\prime }\cdots k_{N}^{\prime }\rangle , \end{array}$$

with ${𝟙}_{M}$ the identity on ${ℋ}^{⊗M}$, the *M*-particle subspace. Because

(A9) $$\begin{array}{c} \sum_{j=1}^{N}{𝟙}_{j-1}⊗P_{S}⊗{𝟙}_{N-j}\equiv \overset{N}{\underset{j=1}{\hat{⨁}}}P_{S}, \end{array}$$

we conclude that

(A10) $$\begin{array}{c} \begin{array}{cc} \{\hat{A},{\hat{N}}_{S}\}=\sum_{\begin{array}{c} k_{1},\cdots ,k_{N}\\ k_{1}^{\prime },\cdots ,k_{N}^{\prime } \end{array}} & \langle \{A,\hat{⊕}P_{S}\}\rangle {\hat{c}}_{k_{1}}^{†}\cdots {\hat{c}}_{k_{N}}^{†}{\hat{c}}_{k_{N}^{\prime }}\cdots {\hat{c}}_{k_{1}^{\prime }}\\ \; & +2:\hat{A}{\hat{N}}_{S}:. \end{array} \end{array}$$

Therefore

(A11) $$\begin{array}{c} Q(\frac{1}{2}\{A,\hat{⊕}P_{S}/N\})=\frac{1}{2}\{\hat{A},{\hat{N}}_{S}/N\}-:\hat{A}{\hat{N}}_{S}/N: \end{array}$$

□

## Appendix B. N-Body Continuity Equation

Here, we show that a continuity equation for the expectation value of the partition holds for an *N*-body observable $\hat{𝒪}$. In the Schrödinger picture,

(A12) $$\begin{array}{cc} \; & \frac{d}{dt}\langle \hat{𝒪}{|}_{S}\rangle =\langle {\hat{J}}_{𝒪}{|}_{S}\rangle +\langle {\hat{\Sigma }}_{𝒪}{|}_{S}\rangle \end{array}$$

(A13) $$\begin{array}{cc} \; & \langle {\hat{J}}_{𝒪}{|}_{S}\rangle =\frac{1}{2}\langle \{\hat{𝒪},\hat{J}{|}_{S}\}\rangle -\langle :\hat{𝒪}\hat{J}{|}_{S}:\rangle \end{array}$$

(A14) $$\begin{array}{cc} \; & \hat{J}{|}_{S}=i[\hat{H},{\hat{N}}_{S}] \end{array}$$

(A15) $$\begin{array}{ccc} \langle {\hat{\Sigma }}_{𝒪}{|}_{S}\rangle & = & \frac{i}{2}\langle \{[\hat{H},\hat{𝒪}],{\hat{N}}_{S}\}\rangle -i\langle :[\hat{H},\hat{𝒪}]{\hat{N}}_{S}:\rangle +\\ & & +\langle (\partial_{t}\hat{𝒪}){|}_{S}\rangle . \end{array}$$

Above, we allow for explicit time dependence in $\hat{H}$ and $\hat{𝒪}$, and the evolution of the system is accounted for in the density matrix. To prove this, we shift Equation (17) into the Schrödinger picture and then apply equation Equation (58). First, recall that for any operator $\hat{𝒪}$ in the Schrödinger picture

(A16) $$\begin{array}{c} \frac{d}{dt}\langle \hat{𝒪}\rangle =i\langle [\hat{H},\hat{𝒪}]\rangle +\langle \partial_{t}\hat{𝒪}\rangle \end{array}$$

Using the identity

(A17) $$\begin{array}{c} \left[A,\{B,C\}]=\{[A,B],C\}+\{B,[A,C]\right\} \end{array}$$

we have that

(A18) $$\begin{array}{c} i[\hat{H},\{\hat{𝒪},{\hat{N}}_{S}\}]=i\{[\hat{H},\hat{𝒪}],{\hat{N}}_{S}\}+\{\hat{𝒪},{\hat{J}}_{S}\}. \end{array}$$

Then, because the commutator of any *N*-body and one-body operator is a normal ordered *N*-body operator, $:[\hat{A},\hat{B}]:=[\hat{A},\hat{B}]$. Therefore, in the absence of interactions,

(A19) $$\begin{array}{c} \begin{array}{cc} \left[\hat{H},:\hat{𝒪}{\hat{N}}_{S}:\right] & =:[\hat{H},:\hat{𝒪}{\hat{N}}_{S}:]:\\ \; & =:[\hat{H},\hat{𝒪}{\hat{N}}_{S}]:\\ \; & =:[\hat{H},\hat{𝒪}]{\hat{N}}_{S}:+:\hat{O}[\hat{H},{\hat{N}}_{S}]: \end{array} \end{array}$$

Equation (A12) then follows immediately from the definition of a partition of an *N*-body observable, Equation (58). Note that this also provides an extension of the continuity equation for time-dependent Hamiltonians.
